# Non-association between low vitamin d levels and aeroallergen-positivity evaluated using multiple allergen simultaneous test in Korean adults

**DOI:** 10.1186/s13223-021-00525-6

**Published:** 2021-02-27

**Authors:** Jee Hye Wee, Sung Woo Cho, Jeong-Whun Kim, Chae-Seo Rhee

**Affiliations:** 1 Department of Otorhinolaryngology-Head and Neck Surgery, Hallym University Sacred Heart Hospital, Hallym University College of Medicine, Anyang, Korea; 2 Department of Otorhinolaryngology-Head and Neck Surgery, Seoul National University Bundang Hospital, Seoul National University College of Medicine, Seongnam, Korea; 3 Sensory Organ Research Institute, Medical Research Center, Seoul National University, Seoul, Korea; 4 Institute of Allergy and Clinical Immunology, Medical Research Center, Seoul National University, Seoul, Korea; 5 Department of Otorhinolaryngology-Head and Neck Surgery, Seoul National University Hospital, Seoul National University College of Medicine, 101 Daehagro, Jongro-gu, Seoul, 03080 Korea

**Keywords:** Vitamin D, Avitaminosis, Allergy and immunology, Population surveillance, Health care surveys

## Abstract

**Background:**

Studies on the association between vitamin D levels and allergen sensitization have reported conflicting results. We aimed to evaluate the association between low vitamin D levels and sensitization to 59 aeroallergens in Korean adults.

**Methods:**

We retrospectively reviewed serum 25-hydroxyvitamin D (25[OH]D) measurements of participants (n = 57,467) in a healthcare center between May 2003 and June 2020. Serum 25(OH)D levels were categorized as follows: severe deficiency (< 10 ng/mL), deficiency (10 to < 20 ng/mL), insufficiency (20 to < 30 ng/mL), and sufficiency (≥ 30 ng/mL). Among all subjects, 1277 simultaneously underwent the multiple allergen simultaneous test. Multiple linear and logistic regression analyses were used to estimate coefficients and odds ratios (ORs) with 95% confidence interval (CI) for the association between serum vitamin D deficiency and aeroallergen sensitization after adjustment for potential confounders. Subgroup analyses were conducted for the types of aeroallergen (house dust mites, pollens, animal dander, foods, cockroach, and fungus).

**Results:**

Vitamin D deficiency, defined as serum 25(OH)D level < 20 ng/mL, was noted in 56.4% of participants. There were significant differences in serum 25(OH)D levels according to sex, age, season, and bone mineral density (all P < 0.001). In multiple linear regression analyses, serum 25(OH)D levels were significantly lower in young subjects (adjusted coefficient [95% CI], 0.188 [0.101, 0.275]) and during winter (− 4.114 [− 6.528, − 1.699]). However, no significant association was observed between serum 25(OH)D levels and allergen sensitization (adjusted coefficients [95% CI], − 0.211 [− 1.989, 1.567], P = 0.816). In multivariate logistic regression analyses, male sex, young age, and winter season were significant risk factors for vitamin D deficiency. However, allergen sensitization showed no significant association with 25(OD)D levels after adjusting for confounders (adjusted OR [95% CI], 1.037 [0.642, 1.674] in insufficiency; 0.910 [0.573, 1.445] in deficiency; 0.869 [0.298, 2.539] in severe deficiency groups, P for trend = 0.334). There were consistent findings across subgroups regarding type of aeroallergen sensitized.

**Conclusion:**

Vitamin D deficiency was prevalent but was not significantly associated with aeroallergen sensitization in Korean adults. To the best of our knowledge, this is the first large-scale study to evaluate the association between vitamin D deficiency and sensitization to 59 different aeroallergens.

## Background

Vitamin D has a unique feature that is synthesized by the skin in response to ultraviolet-B exposure, and its dietary sources are very limited [1]. Changes in indoor lifestyles and air pollution have resulted in an increased number of vitamin D deficiency cases, which has become a global public health problem. Approximately 1 billion people worldwide have vitamin D deficiency (defined as < 20 ng/mL), while 50% of the population has vitamin D insufficiency (defined as 21–29 ng/mL) [2]. According to the data from the Korean National Health and Nutrition Examination Survey (KNHANES) 2008–2014, the prevalence of vitamin D deficiency (defined as < 20 ng/mL) was 51.8% and 68.2% in males and females in 2008, but increased to 75.2% and 82.5% in 2014, respectively [3].

Vitamin D status is generally determined based on serum 25-hydroxyvitamin D (25[OH]D) levels [4]. Although there has been extensive debate on the definition of vitamin D deficiency, vitamin D deficiency is defined as a serum 25(OH)D level of < 50 nmol/L or 20 ng/mL, while insufficiency is defined as a serum 25(OH)D level of 51–74 nmol/L (or 21–29 ng/mL); sufficiency is defined as a serum 25(OH)D level of > 75 nmol/L (or 30 ng/mL), generally [1, 5]. Several studies have reported that vitamin D deficiency is correlated with an increased risk of non-bone-related disorders, such as hypertension, diabetes mellitus, rheumatoid arthritis, and infectious diseases [5–7]. Furthermore, given that vitamin D has gained increasing interest as an immune modulator, it is thought that vitamin D deficiency may be related to allergic diseases [8].

Many observational studies have reported on the relationship between serum 25(OH)D level and allergic diseases, but there are still conflicting results. A study by the KNHANES 2009 reported that subjects with low levels of serum 25(OH)D had a significantly higher risk of allergic rhinitis compared to those with serum 25(OH)D levels of at least 25 ng/mL (serum 25[OH]D lower than 15 ng/mL: hazard ratio, 95% confidence interval [CI], 1.559 [1.099, 2.210]; serum 25[OH]D of at least 15 to lower than 25 ng/mL: 1.430 [1.020, 2.006]) [9]. In contrast, a study conducted in the United States based on the third National Health and Nutrition Examination Survey reported that the prevalence of allergic rhinitis increased with increasing levels of 25(OH)D (odds ratio [OR] [95% CI], 1.25 [1.03, 1.53]) [10]. On the other hand, a meta-analysis showed that the pooled ORs for the incidence of allergic rhinitis according to vitamin D levels were not statistically significant for either children (OR [95% CI], 0.91 [0.41, 2.03]) or adults (1.06 [0.63, 1.77]) [11].

Furthermore, only a few studies have focused on the association between vitamin D levels and allergen sensitization [12–14]. They tested a small number of allergens: 1 allergen in a study conducted by Keet et al. [12], 3 allergens in a study published by Cheng et al. [13], and 17 allergens in a study conducted by Sharief et al. [14]. Moreover, the association of vitamin D levels and allergen sensitization remains controversial. Keet et al. [12] and Cheng et al. [13] reported that there was no association between vitamin D levels and allergen sensitization, whereas Sharief et al. [14] showed that there was some protective association between vitamin D deficiency and allergen sensitization. This study aimed to analyze serum vitamin D levels and to evaluate whether vitamin D deficiency is associated with sensitization to 59 aeroallergens after adjusting for potential confounders in Korean adults.

## Methods

### Study population

We used a clinical data warehouse (CDW) system [15] that was developed to provide big data analytics tools based on in-memory database technology in our hospital. This study was approved by the Institutional Review Board of Seoul National University Bundang Hospital (No. B-2008-630-005). Considering the retrospective study design, the requirement for informed consent was waived. We retrospectively collected data of the serum 25(OH)D measurements of participants (n = 57,467) aged 20 years or older in Seoul National University Bundang Hospital Health Care Center between May 2003 and June 2020.

### Assessment of outcomes and confounders

The enrolled subjects were categorized into five groups according to age in years (20–29, 30–39, 40–49, 50–59, and ≥ 60 years). The seasons during which blood samples were collected were divided into spring (March to May), summer (June to August), fall (September to November), and winter (December to February). Serum 25(OH)D levels were measured and expressed in ng/mL using analysis on liquid chromatography/mass spectrometry. Serum 25(OH)D levels were categorized as follows: severe deficiency (< 10 ng/mL), deficiency (10 to < 20 ng/mL), insufficiency (20 to < 30 ng/mL), and sufficiency (≥ 30 ng/mL) [1, 5]. Histories of diabetes, hypertension, and hyperlipidemia were obtained. Bone mineral density was measured using dual-energy X-ray absorptiometry (DEXA). According to the World Health Organization study group, T-scores at or above − 1.0 are considered normal, those between − 1.0 and − 2.5 are considered as osteopenia, and those at or below − 2.5 are considered as osteoporosis [16].

Among the enrolled study subjects, 1277 participants, who self-selected to have allergen sensitization testing done, simultaneously underwent the multiple allergen simultaneous test (MAST). MAST results were obtained for serum-specific IgE for 59 common aeroallergens (Additional file 1: Table S1). MAST-positive was defined as class 2 (≥ 0.7 IU/mL) or more to an at least one allergen [17, 18]. The 59 aeroallergens were divided into seven allergen groups: house dust mites, pollens, animal dander, foods, cockroach, fungus, and others.

### Statistical analysis

Data of the prevalence of vitamin D deficiency were presented as percentage ± standard deviation (SD) for each group. Student’s *t* test or ANOVA was used to compare the mean serum 25(OH)D levels across the demographic categories. The chi-square test was used to determine whether there were significant differences between the allergen-positive and allergen-negative groups among vitamin D level categories. Multiple linear regression analysis was performed to identify potential confounding variables of serum 25(OH)D levels. The following independent variables were included in the model: age (as a continuous variable), sex, season, bone mineral density, medical diseases, and allergen sensitization (as categorical variables).

Multivariate logistic regression models were also created to calculate the adjusted ORs and their 95% CIs of the risk factors on comparing 25(OH)D levels of < 10 ng/mL, 10 to < 20 ng/mL, and 20 to < 30 ng/mL with 25(OH)D levels of ≥ 30 ng/mL. A P-value of less than 0.05 was considered to indicate statistical significance. Statistical analyses were conducted using SPSS complex version 22.0 (IBM, Armonk, NY, USA).

## Results

Serum 25(OH)D levels according to subject characteristics are presented in Table 1. The mean serum 25(OH)D level was lower in men (20.0 ± 8.3 ng/mL) than in women (20.4 ± 10.5 ng/mL) (P < 0.001). Participants aged < 30 years had the lowest serum 25(OH)D levels (16.0 ± 7.3 ng/mL), whereas those aged ≥ 60 years had the highest serum 25(OH)D levels (23.6 ± 10.5 ng/mL); this difference was statistically significant (P < 0.001). Serum 25(OH)D levels were observed to be lower in the winter (18.9 ± 9.7 ng/mL) than in the summer (21.2 ± 8.5 ng/mL) (P < 0.001). The mean serum 25(OH)D level was significantly lower in participants with a normal T-score in DEXA (19.5 ± 8.9 ng/mL) than in those with a decreased T-score (21.2 ± 10.1 ng/mL in osteopenia; 23.0 ± 10.6 ng/mL in osteoporosis; P < 0.001). However, mean serum 25(OH)D levels did not show significant differences between the MAST-positive (n = 617, 21.1 ± 9.2 ng/mL) and MAST-negative (n = 660, 21.7 ± 9.2 ng/mL) groups (P = 0.265). Serum 25(OH)D levels by the characteristics of 1277 participants who had undergone MAST showed consistent results (Additional file 1: Table S2).

**Table 1:** The serum levels of 25(OH)D by the characteristics of participants

	**No.**	**Serum 25(OH)D level** **(mean ± SD, ng/mL)**	***P-value***
Overall	57,467	20.2 ± 9.3	
Sex			
Male	31,520	20.0 ± 8.3	< 0.001^*^
Female	25,947	20.4 ± 10.5	
Age group			
20–29	2674	16.0 ± 7.3	< 0.001^†^
30–39	6796	17.2 ± 7.7	
40–49	14,543	18.3 ± 8.0	
50–59	19,569	20.8 ± 9.2	
≥ 60	13,885	23.6 ± 10.5	
Season			
Spring	14,565	19.6 ± 9.9	< 0.001^†^
Summer	14,671	21.2 ± 8.5	
Fall	13,555	21.1 ± 8.9	
Winter	14,676	18.9 ± 9.7	
DEXA			< 0.001^†^
Normal	15,437	19.5 ± 8.9	
Osteopenia	8048	21.2 ± 10.1	
Osteoporosis	1762	23.0 ± 10.6	
Missing	32,220		
MAST			0.265
Positive	617	21.1 ± 9.2	
Negative	660	21.7 ± 8.2	
Missing	56,190		

*SD* standard deviation, *DEXA* Dual energy X-ray absorptiometry, *MAST* multiple allergen simultaneous test

^*^Student’s *t* test, ^†^ANOVA test, with *P* < 0.05 considered significant

**Fig. 1:**
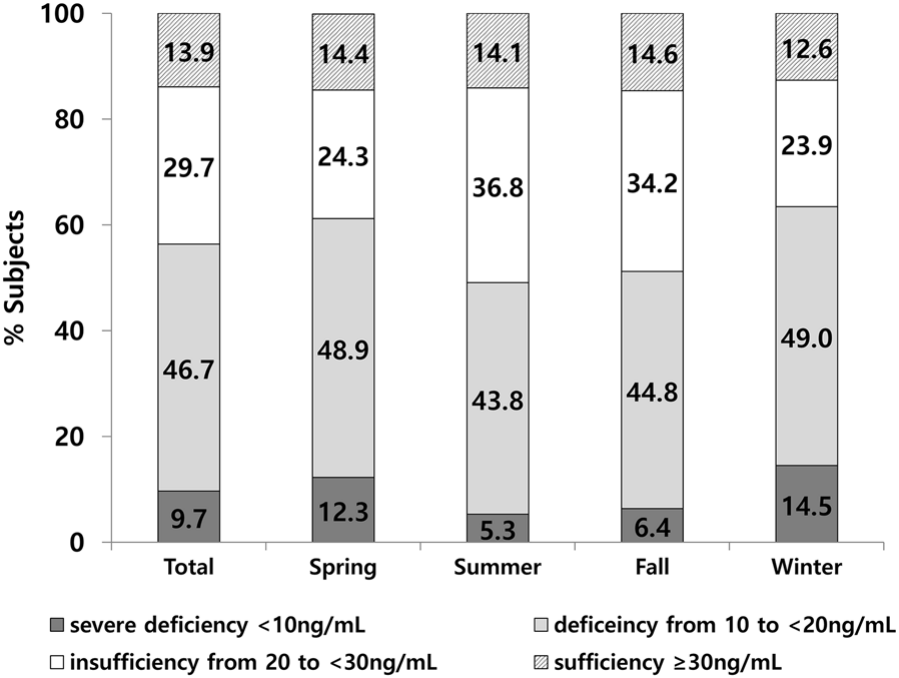
The percentage of participants with serum 25(OH)D levels of < 10 ng/mL, 10 to < 20 ng/mL, and 20 to < 30 ng/mL and ≥ 30 ng/mL according to the season

**Fig. 2:**
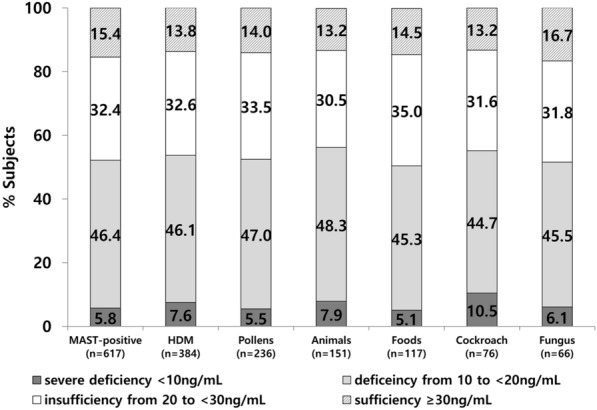
The percentage of MAST-positive participants with serum 25(OH)D levels of < 10 ng/mL, 10 to < 20 ng/mL, and 20 to < 30 ng/mL and ≥ 30 ng/mL according to the types of aeroallergen sensitized

The mean 25(OH)D level was 20.2 ± 9.3 ng/mL (range: 2.0–141.8 ng/mL), and 9.7%, 56.4%, and 86.1% of the subjects had 25(OH)D levels of < 10 ng/mL, < 20 ng/mL, and < 30 ng/mL, respectively (Fig. 1). Figure 1 shows the prevalence of vitamin D deficiency according to the seasons. Even in the summer, almost half of the subjects (49.1%) showed vitamin D deficiency (< 20 ng/mL). Figure 2 shows the proportion of vitamin D level categories in MAST-positive participants according to the types of aeroallergen sensitized. More than half of the subjects had vitamin D deficiency (< 20 ng/mL) in all subgroups. There was no significant difference between the MAST-positive and MAST-negative groups in vitamin D level categories (P = 0.441) and there was also no significant difference when analyzed by each allergen type (all P > 0.05, Table 2). In addition, among the 617 subjects (MAST-positive), 340 patients (55.1%) had a single category of aeroallergen sensitization and 277 (44.9%) patients had multiple categories of sensitization, showing no significant difference between the two groups in vitamin D level categories (P = 0.505).

**Table 2:** Percentage of serum 25(OH)D levels of < 10 ng/mL, 10 to < 20 ng/mL, 20 to < 30 ng/mL, and ≥ 30 ng/mL according to the allergen sensitization

**Variables**	**Participants (n, %)**				P-value*
	Serum 25(OH)D ≥ 30	20 ≤ Serum 25(OH)D < 30	10 ≤ Serum 25(OH)D < 20	Serum 25(OH)D < 10	
MAST					0.441
Positive (n = 617)	95 (15.4)	200 (32.4)	286 (46.4)	36 (5.8)	
Negative (n = 660)	118 (17.9)	220 (33.3)	279 (42.3)	43 (6.5)	
House dust mites					0.185
Positive (n = 384)	53 (13.8)	125 (32.6)	177 (44.2)	29 (6.2)	
Negative (n = 893)	160 (17.9)	295 (33.0)	388 (43.4)	50 (5.6)	
Pollens					0.569
Positive (n = 236)	33 (14.0)	79 (33.5)	111 (47.0)	13 (5.5)	
Negative (n = 1041)	180 (17.3)	341 (32.8)	454 (43.6)	66 (6.3)	
Animal dander					0.388
Positive (n = 151)	20 (13.2)	46 (30.5)	73 (48.3)	12 (7.9)	
Negative (n = 1126)	193 (17.1)	374 (33.2)	492 (43.7)	67 (6.0)	
Foods					0.849
Positive (n = 117)	17 (14.5)	41 (35.0)	53 (45.3)	6 (5.1)	
Negative (n = 1160)	196 (16.9)	379 (32.7)	512 (44.1)	73 (6.3)	
Cockroach					0.375
Positive (n = 76)	10 (13.2)	24 (31.6)	34 (44.7)	8 (10.5)	
Negative (n = 1201)	203 (16.9)	396 (33.0)	531 (44.2)	71 (5.9)	
Fungus					0.997
Positive (n = 66)	11 (16.7)	21 (31.8)	30 (45.5)	4 (6.1)	
Negative (n = 1211)	202 (16.7)	399 (32.9)	535 (44.2)	75 (6.2)	

MAST: multiple allergen simultaneous test

*Chi-square test, with *P* < 0.05 considered significant

On multiple linear regression analysis, serum 25(OH)D levels were significantly lower in young subjects (adjusted coefficients [95% CI], 0.188 [0.101, 0.275], P < 0.001) and during winter (–4.114 [–6.528, –1.699], P = 0.001) (Table 3). However, no significant association was observed between serum 25(OH)D levels and MAST-positivity after adjusting for confounders such as sex, age, season, and medical diseases including osteopenia and osteoporosis (adjusted coefficient [95% CI], –0.211 [–1.989, 1.567], P = 0.816). When stratified by types of aeroallergen sensitized, there was also no significant association (all P > 0.05, Table 4).

**Table 3:** Results of a multiple linear regression analysis on variables of vitamin D levels

Variables	Adjusted coefficients (SD)	95% CI	*P*-value
Sex (male)	− 0.637 (0.915)	− 2.437 to 1.162	0.486
Age	0.188 (0.915)	0.101 to 0.275	< 0.001*
Season			
Spring	− 2.113 (1.248)	− 4.567 to − 0.341	0.091
Summer	Reference		
Fall	0.098 (1.274)	− 2.409 to 2.605	0.939
Winter	− 4.114 (1.227)	− 6.528 to − 1.699	0.001*
DEXA			
Normal	Reference		
Osteopenia	0.364 (0.963)	− 1.531 to 2.259	0.706
Osteoporosis	1.043 (1.871)	− 2.639 to 4.725	0.578
Diabetes mellitus	− 0.764 (1.160)	− 3.046 to 1.519	0.511
Hypertension	− 0.271 (0.543)	− 3.046 to 1.519	0.618
Hypercholesterolemia	− 0.164 (0.395)	− 0.613 to 0.941	0.678
MAST (Positive)	− 0.211 (0.904)	− 1.989 to 1.567	0.816

*SD* standard deviation, *CI* confidence interval, *DEXA* dual energy x-ray absorptiometry, *MAST* multiple allergen simultaneous test

*Multiple linear regression analysis, with *P* < 0.05 considered significant

**Table 4:** Associations between allergen sensitization and serum 25(OH)D levels according to types of aeroallergen

Types of aeroallergen	Adjusted coefficients (SD)	95% CI	*P*-value*
House dust mites (n = 384)	0.271 (1.069)	− 1.831 to 2.374	0.800
Pollens (n = 236)	− 0.175 (0.569)	− 1.294 to 0.943	0.758
Animal dander (n = 151)	0.308 (0.528)	− 0.731 to 1.346	0.560
Foods (n = 117)	0.173 (0.329)	− 0.474 to 0.821	0.599
Cockroach (n = 76)	0.054 (0.391)	− 0.715 to 0.822	0.891
Fungus (n = 66)	0.356 (0.470)	− 0.568 to 1.280	0.449

*SD* standard deviation, *CI* confidence interval

*Multiple linear regression analysis, adjusted for age, sex, season, and medical diseases (osteopenia, osteoporosis, diabetes mellitus, hypertension, and hypercholesterolemia), with *P* < 0.05 considered significant

Tables 5 shows adjusted ORs for the associations between risk factors and different serum 25(OH)D levels. Multiple logistic regression analyses after adjusting for variables showed the following significant risk factors for vitamin D deficiency: male sex (P for trend < 0.001), young age (P for trend < 0.001), and winter season (P for trend = 0.002). However, no significant association was observed between MAST-positivity and vitamin D deficiency (adjusted OR [95% CI], 1.037 [0.642, 1.674], P = 0.882 in insufficiency; 0.910 [0.573, 1.445], P = 0.689 in deficiency; 0.869 [0.298, 2.539], P = 0.148 in severe deficiency groups; P for trend = 0.334). In subgroup analysis according to the types of aeroallergen sensitized, there was no significant association between allergen sensitization and vitamin D deficiency (all P > 0.05, Table 6).

**Table 5:** Adjusted odds ratios for the association between risk factors and serum 25(OH)D levels of < 10 ng/mL, 10 to < 20 ng/mL, and 20 to < 30 ng/mL compared with the reference group (≥ 30 ng/mL)

Variables	Adjusted ORs (95% confidence interval)				*P* for trend
	Serum 25(OH)D ≥ 30	20 ≤ Serum 25(OH)D < 30	10 ≤ Serum 25(OH)D < 20	Serum 25(OH)D < 10	
Sex (male)	1	2.212 (1.351–3.622) ^*^	1.937 (1.204–3.117) ^*^	0.470 (0.170–1.300)	< 0.001
Age group					< 0.001
20–29 vs ≥ 60	1	N/A	2.171 (0.199–23.697)	N/A	
30–39 vs ≥ 60	1	3.656 (1.070–12.491) ^*^	4.686 (1.422–15.441) ^*^	11.736 (2.124–64.850) ^*^	
40–49 vs ≥ 60	1	4.496 (1.775–11.387) ^*^	7.627 (3.135–18.557) ^*^	13.978 (3.449–56.660) ^*^	
50–59 vs ≥ 60	1	3.187 (1.844–5.509) ^*^	4.376 (2.577–7.431) ^*^	5.868 (1.970–17.477) ^*^	
Season					0.002
Spring vs Winter	1	0.428 (0.223–0.821) ^*^	0.510 (0.279–0.933)	0.423 (0.160–1.114)	
Summer vs Winter	1	0.922 (0.470–1.809)	0.621 (0.321–1.202)	0.149 (0.037–0.599)^*^	
Fall vs Winter	1	1.065 (0.540–2.103)	0.676 (0.347–1.320)	0.235 (0.065–0.844)^*^	
DEXA					0.274
Normal vs Osteoporosis	1	1.037 (0.460–2.337)	1.770 (0.766–4.093)	N/A	
Osteopenia vs Osteoporosis	1	0.795 (0.341–1.853)	1.150 (0.481–2.751)	N/A	
Diabetes mellitus	1	0.782 (0.420–1.458)	1.371 (0.770–2.442)	0.804 (0.211–3.066)	0.172
Hypertension	1	1.311 (0.755–2.275)	1.090 (0.638–1.860)	1.022 (0.333–3.135)	0.777
Hypercholesterolemia	1	0.741 (0.408–1.347)	0.834 (0.471–1.477)	0.435 (0.090–2.094)	0.615
MAST (Positive)	1	1.037 (0.642–1.674)	0.910 (0.573–1.445)	0.869 (0.298–2.539)	0.334

*DEXA* dual energy X-ray absorptiometry, *MAST* multiple allergen simultaneous test

^*^Multivariate logistic regression analyses, with *P* < 0.05 considered significant

**Table 6:** Adjusted odds ratios for the association between aeroallergen sensitization and serum 25(OH)D levels of < 10 ng/mL, 10 to < 20 ng/mL, and 20 to < 30 ng/mL compared with the reference group (≥ 30 ng/mL) according to the types of aeroallergen sensitization

Types of aeroallergen	Adjusted ORs (95% confidence interval)				*P* for trend
	Serum 25(OH)D ≥ 30	20 ≤ Serum 25(OH)D < 30	10 ≤ Serum 25(OH)D < 20	Serum 25(OH)D < 10	
House dust mites (n = 384)	1	0.973 (0.566–1.671)	0.977 (0.577–1.652)	0.728 (0.273–1.942)	0.930
Pollens (n = 236)	1	0.871 (0.479–1.583)	0.978 (0.553–1.731)	0.663 (0.204–2.154)	0.864
Animal dander (n = 151)	1	0.626 (0.290–1.353)	0.691 (0.335–1.422)	0.315 (0.063–1.578)	0.426
Foods (n = 117)	1	0.791 (0.381–1.641)	0.622 (0.305–1.268)	0.193 (0.023–1.605)	0.244
Cockroach (n = 76)	1	0.712 (0.276–1.839)	0.536 (0.208–1.379)	0.541 (0.057–5.114)	0.631
Fungus (n = 66)	1	0.823 (0.294–2.300)	0.790 (0.289–2.160)	N/A	0.272

^*^Multivariate logistic regression analyses, adjusted with sex, age group, season, and medical diseases (osteopenia, osteoporosis, diabetes mellitus, hypertension, and hypercholesterolemia) with *P* < 0.05 considered significant

## Discussion

To analyze the association between allergen sensitization and low vitamin D levels, we determined the serum-specific IgE levels for 59 common airborne indoor and outdoor allergens. We could not observe any significant association between serum vitamin D levels and allergen sensitization. There were consistent findings across subgroups regarding the types of aeroallergen sensitized. To the best of our knowledge, this is the first large-scale study to evaluate the association between vitamin D deficiency and sensitization to 59 different aeroallergens.

Only few studies have been conducted on the association between vitamin D levels and allergen sensitization, and the relationship is still unclear. A cohort study that included 260 subjects in the U.S. reported that serum vitamin D levels were not associated with incident mouse sensitization (OR [95% CI], 3.2 [0.7, 14.1], P = 0.13) [12]. A cross-sectional population-based study that used data from the KNHANES 2008–2010 showed no association between serum vitamin D levels and sensitization to three allergens sensitization (*Dermatophagoides farinae*: OR [95% CI], 0.86 [0.61, 1.21]; cockroaches: 1.25 [0.75. 2.06]; and dogs: 1.58 [0.80, 3.11]) [13]. These findings are consistent with those observed in our study, although they tested a small number of allergens. In contrast, a large cross-sectional study in the U.S. found a significant association between serum vitamin D levels and allergen sensitization in three out of 11 allergens in adults [14]. They showed that 25(OH)D levels of 15–29 ng/mL had protective associations with sensitization to dog (OR [95% CI], 0.71 [0.53, 0.96]) and cockroach allergens (OR [95% CI] 0.64 [0.43, 0.94]), and levels of less than 15 ng/mL showed a protective association with ragweed allergy (OR [95% CI], 0.60 [0.40, 0.89]) [14]. However, in all subgroups according to the types of aeroallergen sensitized, we found no correlation between vitamin D deficiency and allergen sensitization.

The results suggesting that there is no association between vitamin D levels and allergen sensitization in adults can be explained by several factors. First, if the allergies have begun in childhood, then the levels of serum 25(OH)D in adults may not reflect the 25(OH)D status at the time of allergen sensitization. A meta-analysis showed that the associations between vitamin D deficiency and allergen sensitization were seen in children and adolescents but not in adults [19]. Second, it has been speculated that the role of vitamin D in allergen sensitization depends on ethnicity. A previous study showed that specific genotypes modify the association between vitamin D deficiency and allergy risk in adults [20]. Third, expression of some immune system factors involved in allergen sensitization has been shown to be age-dependent. Vitamin D regulates the expression of pattern recognition receptors, such as CD14 [21]. CD14 polymorphism and serum CD14 levels are related to allergen sensitization and are age-specific, which are present during mid-childhood but not in early adulthood [22, 23]. However, further studies are needed to determine the precise role of vitamin D in the pathophysiology of allergen sensitization.

Previous studies have suggested a link between allergic disease/allergen sensitization and low vitamin D level, and recommendations for vitamin D supplementation have also been strengthened. However, the preventive or therapeutic effect of vitamin D supplementation on allergic disease remains controversial. In a birth cohort study from the United States, there was no association between maternal intake of vitamin D during pregnancy and allergen sensitization and allergic rhinitis at school age [24]. In cohort studies conducted in Finland and Germany, a significantly higher rate of allergen sensitization and allergic rhinitis at 31 years of age was observed in a group that had regularly consumed vitamin D supplements in infancy [25], and higher maternal 25(OH)D concentration was associated with an increased risk of food allergen sensitization at the age of 2 years [26]. In contrast, a randomized double-blind placebo-controlled study showed that vitamin D supplementation along with antihistamines resulted in relative symptom improvement in allergic rhinitis patients with vitamin D deficiency. Based on these findings, it is unclear whether vitamin D supplementation should be recommended. Moreover, in the present study, vitamin D deficiency was prevalent, even in the summer, but was not associated with an increased risk of aeroallergen sensitization.

Our study showed that serum vitamin D levels were significantly lower in males and young subjects than in females and older subjects among Korean adults. Globally, vitamin D deficiency is known to be more prevalent in older age groups and to have no significant sex-related differences [6, 27]. Although we cannot state the exact cause of these conflicting results, one possible cause is regional difference. A recent systematic review about vitamin D status in the population reported differences according to regions, with vitamin D levels being significantly higher in North America than in Europe or the Middle East/Africa region [6]. In the Asia/Pacific region, 25(OH)D values were lower in children/adolescents than in adults/elderly people; furthermore, females tended to have significantly low 25(OH)D values [6]. A recent study based on the KNHANES 2008–2014 showed a significantly increasing trend in vitamin D deficiency and showed that vitamin D deficiency was prevalent in young adults, in females, and in the winter [3].

The definition of vitamin D deficiency has been under extensive debate. Unlike the commonly used definition for vitamin D deficiency, serum 25(OH)D levels can be divided into adequate (≥ 50 nmol/L or 20 ng/mL), inadequate (30 to < 50 nmol/L or 12 to < 20 ng/mL), and deficient (< 30 nmol/L or 12 ng/mL) according to the Institute of Medicine guidelines [28]. Current evidence-based consensus guidelines have not been established, and this is of concern because individuals with serum 25(OH)D levels ranging from 20 to < 30 ng/mL may be classified to have insufficiency or adequate status. In the present study, vitamin D insufficiency (serum 25[OH]D level of < 30 ng/mL) was found in most (86.1%) Korean adults. A higher threshold level of serum 25(OH)D would be expected to artificially increase the incidence of vitamin D deficiency. Furthermore, this definition is based on Western populations, and racial/genetic differences could affect the adequate vitamin D level. As there will be differences in vitamin D levels by race/ethnicity, we believe that this Asian study on vitamin D levels, conducted within a single ethnic group, is important, although we could not analyze the results according to race/ethnicity. Additional studies will be needed to evaluate the exact range of vitamin D levels for maintaining bone health and avoiding non-bone-related disorders, including allergic disease, according to sex, age, season, and region in Korea.

The present study has some limitations. First, because it was a cross-sectional study, it was not possible to determine a clear causal relationship between risk factors and vitamin D deficiency and to evaluate the historical levels of vitamin D. Second, we could not perform assessments for allergic rhinitis and asthma. In addition, we did not obtain information about medications including vitamin D supplementation. The serum 25(OH)D levels of subjects with osteopenia or osteoporosis were higher than those of normal subjects, suggesting that participants may have been taking vitamin D supplementation. Lastly, the number of subjects who underwent allergic tests (MAST) was small, compared to the total number of participants included. The possibility of biased results cannot be overlooked, as the participants who visited a health screening center have decided whether to test for allergen sensitization. We used the conventional cutoff level of class 2 as the minimal positive result criteria, but the use of class 1 as a cutoff level could result in an additional 532 patients being included in the MAST-positive group (Additional file 1, Table S3). However, there was no significant difference in serum 25(OH)D levels between the three groups.

Despite these limitations, a potential strength of the present study was that it enrolled a large number of subjects using the CDW system and analyzed a wide array of aeroallergen (59) compared to previous studies. Further studies on a larger general population who had undergone allergic testing could show more clinically significant results.

## Conclusions

Vitamin D deficiency was not associated with an increased risk of allergen sensitization among Korean adults. It is unclear whether vitamin D supplementation should be recommended for prevention of allergic disease. Furthermore, vitamin D deficiency was prevalent, even in the summer, and was more common in Korean males and young individuals. Additional studies are needed to elucidate the adequate vitamin D levels according to sex, age, and racial/genetic differences.

## Supplementary Information

**Additional file 1: Table S1.** Fifty-nine aeroallergens tested by MAST. **Table S2.** The serum levels of 25(OH)D by the characteristics of 1277 participants who had MAST done. **Table S3** The serum levels of 25(OH)D according to the results of MAST.

## Data Availability

The datasets used and/or analyzed during the current study are available from the corresponding author on reasonable request.

## Abbreviations

KNHANES: Korean National Health and Nutrition Examination Survey
25[OH]D: 25-Hydroxyvitamin D
CI: Confidence interval
OR: Odds ratio
CDW: Clinical data warehouse
DEXA: Dual energy X-ray absorptiometry
MAST: Multiple allergen simultaneous test
SD: Standard deviation

## References

[1] Holick MF (2007). Vitamin D deficiency. N Engl J Med.

[2] Nair R, Maseeh A (2012). Vitamin D: The “sunshine” vitamin. J Pharmacol Pharmacother.

[3] Park JH, Hong IY, Chung JW, Choi HS (2018). Vitamin D status in South Korean population: Seven-year trend from the KNHANES. Medicine.

[4] Holick MF (2009). Vitamin D status: measurement, interpretation, and clinical application. Ann Epidemiol.

[5] Holick MF, Binkley NC, Bischoff-Ferrari HA, Gordon CM, Hanley DA, Heaney RP (2011). Evaluation, treatment, and prevention of vitamin D deficiency: an Endocrine Society clinical practice guideline. J Clin Endocrinol Metab.

[6] Hilger J, Friedel A, Herr R, Rausch T, Roos F, Wahl DA (2014). A systematic review of vitamin D status in populations worldwide. Br J Nutr.

[7] Holick MF (2004). Sunlight and vitamin D for bone health and prevention of autoimmune diseases, cancers, and cardiovascular disease. Am J Clin Nutr.

[8] Litonjua AA, Weiss ST (2007). Is vitamin D deficiency to blame for the asthma epidemic?. J Allergy Clin Immunol.

[9] Jung J-W, Kim J-Y, Cho S-H, Choi B-W, Min K-U, Kang H-R (2013). Allergic rhinitis and serum 25-hydroxyvitamin D level in Korean adults. Ann Allergy Asthma Immunol.

[10] Wjst M, Hypponen E (2007). Vitamin D serum levels and allergic rhinitis. Allergy.

[11] Kim YH, Kim KW, Kim MJ, Sol IS, Yoon SH, Ahn HS (2016). Vitamin D levels in allergic rhinitis: a systematic review and meta-analysis. Pediatr Allergy Immunol.

[12] Keet CA, Shreffler WG, Peng RD, Matsui W, Matsui EC (2014). Associations between serum folate and vitamin D levels and incident mouse sensitization in adults. J Allergy Clin Immunol.

[13] Cheng HM, Kim S, Park G-H, Chang SE, Bang S, Won CH (2014). Low vitamin D levels are associated with atopic dermatitis, but not allergic rhinitis, asthma, or IgE sensitization, in the adult Korean population. J Allergy Clin Immunol.

[14] Sharief S, Jariwala S, Kumar J, Muntner P, Melamed ML (2011). Vitamin D levels and food and environmental allergies in the United States: results from the National Health and Nutrition Examination Survey 2005–2006. J Allergy Clin Immunol.

[15] Yoo S, Lee KH, Lee HJ, Ha K, Lim C, Chin HJ (2012). Seoul National University Bundang Hospital's Electronic System for Total Care. Healthcare Inform Res.

[16] WHO Study Group. Assessment of fracture risk and its application to screening for postmenopausal osteoporosis. Report of a WHO Study Group. World Health Organization Technical Report Series. 1994;843:1–129. 7941614

[17] Rim JH, Park BG, Kim JH, Kim HS (2016). Comparison and clinical utility evaluation of four multiple allergen simultaneous tests including two newly introduced fully automated analyzers. Pract Lab Med.

[18] Arbes SJ, Gergen PJ, Vaughn B, Zeldin DC (2007). Asthma cases attributable to atopy: results from the Third National Health and Nutrition Examination Survey. J Allergy Clin Immunol.

[19] Aryan Z, Rezaei N, Camargo CA (2017). Vitamin D status, aeroallergen sensitization, and allergic rhinitis: a systematic review and meta-analysis. Int Rev Immunol.

[20] Vimaleswaran KS, Cavadino A, Hyppönen E (2012). Evidence for a genetic interaction in allergy-related responsiveness to vitamin D deficiency. Allergy.

[21] Sadeghi K, Wessner B, Laggner U, Ploder M, Tamandl D, Friedl J (2006). Vitamin D3 down-regulates monocyte TLR expression and triggers hyporesponsiveness to pathogen-associated molecular patterns. Eur J Immunol.

[22] O'Donnell AR, Toelle BG, Marks GB, Hayden CM, Laing IA, Peat JK (2004). Age-specific relationship between CD14 and atopy in a cohort assessed from age 8 to 25 years. Am J Respir Crit Care Med.

[23] Munthe-Kaas MC, Torjussen TM, Gervin K, Carlsen KCL, Carlsen KH, Granum B (2010). CD14 polymorphisms and serum CD14 levels through childhood: a role for gene methylation?. J Allergy Clin Immunol.

[24] Bunyavanich S, Rifas-Shiman SL, Platts-Mills TA, Workman L, Sordillo JE, Camargo CA (2016). Prenatal, perinatal, and childhood vitamin D exposure and their association with childhood allergic rhinitis and allergic sensitization. J Allergy Clin Immunol.

[25] Hyppönen E, Sovio U, Wjst M, Patel S, Pekkanen J, Hartikainen AL (2004). Infant vitamin d supplementation and allergic conditions in adulthood: northern Finland birth cohort 1966. Ann N Y Acad Sci.

[26] Weisse K, Winkler S, Hirche F, Herberth G, Hinz D, Bauer M (2013). Maternal and newborn vitamin D status and its impact on food allergy development in the German LINA cohort study. Allergy.

[27] Bettencourt A, Boleixa D, Reis J, Oliveira JC, Mendonca D, Costa PP (2018). Serum 25-hydroxyvitamin D levels in a healthy population from the North of Portugal. J Steroid Biochem Mol Biol.

[28] Ross AC, Manson JE, Abrams SA, Aloia JF, Brannon PM, Clinton SK (2011). The 2011 report on dietary reference intakes for calcium and vitamin D from the Institute of Medicine: what clinicians need to know. J Clin Endocrinol Metab.

