# Supplementary material for: Non-association between low vitamin d levels and aeroallergen-positivity evaluated using multiple allergen simultaneous test in Korean adults
Source: Allergy Asthma Clin Immunol. 2021 Feb 27;17:23. doi: 10.1186/s13223-021-00525-6 (PMC7913436; doi:10.1186/s13223-021-00525-6)
Supplement: Supplementary file 1 — Additional file 1: Table S1. Fifty-nine aeroallergens tested by MAST. Table S2. The serum levels of 25(OH)D by the characteristics of 1277 participants who had MAST done. Table S3 The serum levels of 25(OH)D according to the results of MAST. [file 13223_2021_525_MOESM1_ESM.docx]

**Table S1.** Fifty-nine aeroallergens tested by MAST.

| 1. House dust | 31. Sweet vernal/Orch |
| --- | --- |
| 2. *Dermatophagoides farinae* | 32. Bermuda grass |
| 3. *Dermatophagoides pteronyssinus* | 33. Timothy grass |
| 4. Cat | 34. *Penicillium notatum* |
| 5. Dog | 35. *Candida albicans* |
| 6. Egg white | 36. Hazel |
| 7. Milk | 37. Olive |
| 8. Cockroach | 38. Maple leaf sycamo |
| 9. Peanut | 39. Wilow |
| 10. Soy bean | 40. Cottonwood |
| 11. Wheat | 41. White ash |
| 12. Alder | 42. White pine |
| 13. Birch | 43. Japanese cedar |
| 14. Oak | 44. Acacia |
| 15. Common ragweed | 45. Ox-eye daisy |
| 16. Japanese hop | 46. Dandelion |
| 17. Mugwort | 47. Plantain |
| 18. *Alternaria alternata* | 48. Russian thistle |
| 19. *Cladosporium herbarum* | 49. Goldenrod |
| 20. *Aspergillus fumigatus* | 50. Common pigweed |
| 21. Crab | 51. Hevea latex |
| 22. Shrimp | 52. Bee venom |
| 23. Mackerel | 53. Wasp venom |
| 24. Cultivated rye | 54. Mouse/Rat |
| 25. CCD (Cross-reactive carbohydrate determinant) | 55. Rabbit |
| 26. Peach | 56. Guniea pig |
| 27. Apple | 57. Wool, sheep |
| 28. Seasme | 58. Hamster |
| 29. Acarus siro | 59. Horse |
| 30. *Tyrophagus putrescentiae* |  |

MAST: multiple allergen simultaneous test

**Table S2.** The serum levels of 25(OH)D by the characteristics of 1277 participants who had MAST done.

|  | **No.** | **Serum 25(OH)D level**  **(mean ± SD, ng/mL)** | ***P*-value** |
| --- | --- | --- | --- |
| **Overall** | 1277 | 21.4 ± 9.2 |  |
| **Sex** |  |  |  |
| Male | 791 | 20.6 ± 8.0 | < 0.001^*^ |
| Female | 486 | 22.6 ± 10.8 |  |
| **Age group** |  |  |  |
| 20 – 29 | 85 | 16.2 ± 5.7 | < 0.001^†^ |
| 30 – 39 | 113 | 18.2 ± 7.3 |  |
| 40 – 49 | 230 | 19.0 ± 7.8 |  |
| 50 – 59 | 417 | 20.7 ± 8.3 |  |
| ≥ 60 | 432 | 25.2 ± 9.2 |  |
| **Season** |  |  |  |
| Spring | 348 | 21.6 ± 10.0 | 0.001^†^ |
| Summer | 304 | 22.2 ± 7.9 |  |
| Fall | 270 | 22.5 ± 8.9 |  |
| Winter | 355 | 19.7 ± 9.6 |  |
| **DEXA** |  |  | 0.003^†^ |
| Normal | 390 | 21.6 ± 9.0 |  |
| Osteopenia | 194 | 23.2 ± 10.2 |  |
| Osteoporosis | 46 | 26.1 ± 10.6 |  |
| Missing | 647 |  |  |
| **MAST** |  |  | 0.226 |
| Positive | 617 | 21.1 ± 9.2 |  |
| Negative | 660 | 21.7 ± 9.2 |  |

*SD* standard deviation, *DEXA* Dual energy x-ray absorptiometry, *MAST* multiple allergen simultaneous test.

^*^Student’s *t* test, ^†^ANOVA test, with *P* < 0.05 considered significant.

**Table S3** The serum levels of 25(OH)D according to the results of MAST.

|  | **No.** | **Serum 25(OH)D level**  **(mean ± SD, range, ng/mL)** | ***P*-value*** |
| --- | --- | --- | --- |
| **MAST** |  |  | 0.538 |
| ≥ Class 2 | 617 | 21.1 ± 9.2 (3.2–68.0) |  |
| Class 1 | 532 | 21.7 ± 9.4 (5.1–51.1) |  |
| Negative | 128 | 21.7 ± 8.2 (6.7–42.4) |  |

MAST: multiple allergen simultaneous test, SD: standard deviation

*ANOVA test, with P < 0.05 considered significant
